# Identification of myocilin as a blood plasma protein and analysis of its role in leukocyte adhesion to endothelial cell monolayers

**DOI:** 10.1371/journal.pone.0209364

**Published:** 2018-12-17

**Authors:** José-Daniel Aroca-Aguilar, Ana Fernández-Navarro, Jesús Ontañón, Miguel Coca-Prados, Julio Escribano

**Affiliations:** 1 Laboratorio de Genética Molecular Humana, Facultad de Medicina/Instituto de Investigación en Discapacidades Neurológicas (IDINE), Universidad de Castilla-La Mancha, Albacete, Spain; 2 Cooperative Research Network on Prevention, Early Detection and Treatment of Prevalent Degenerative and Chronic Ocular Pathology (OftaRed), Instituto de Salud Carlos III, Madrid, Spain; 3 Servicio de Inmunología, Complejo Hospitalario Universitario de Albacete, Castilla la Mancha, Spain; 4 Department of Ophthalmology and Visual Science, Yale University School of Medicine, New Haven, CT, United States of America; 5 Fundación de Investigación Oftalmológica Instituto Oftalmológico Fernández-Vega, Oviedo, Spain; University of Iowa, UNITED STATES

## Abstract

Myocilin is an extracellular glycoprotein with a poorly understood biological function and typically known because of its association with glaucoma. In this study, we analyzed the expression and biological activity of human myocilin in some non-ocular tissues. Western immunoblot showed the presence of myocilin in blood plasma as well as in liver and lymphoid tissues (thymus and lymph node). Quantitative PCR confirmed the expression of *MYOC* in these lymphoid organs and revealed that its mRNA is also present in T-lymphocytes and leukocytes. In addition, detection of 30 kDa C-terminal myocilin fragments in thymus and liver suggested that myocilin undergoes an *in vivo* proteolytic processing that might regulate its biological activity. The presence of myocilin in blood was further corroborated by peptide mass fingerprinting of the HPLC-isolated protein, and gross estimation of its concentration by Western immunoblot indicated that it is a medium-abundance serum protein with an approximate concentration of 0.85 mg/ml (15.5 μM). Finally, *in vitro* analyses indicated that myocilin acts as an anti-adhesive protein for human circulating leukocytes incubated with endothelial cell monolayers. Altogether, these data provide insightful information on new biological properties of myocilin and suggest its putative role as a blood matricellular protein.

## Introduction

After 20 years of intense myocilin study, its biological function remains elusive. Independent isolation of expressed sequence tags from subtracted ciliary body [1, 2] and retina [3] cDNA libraries revealed the expression of the myocilin gene in ocular tissues. The protein, initially called *Trabecular Meshwork Inducible Glucocorticoid Response* (*TIGR*), was identified in human trabecular meshwork cell cultures after prolonged exposure to glucocorticoids [4], and was later denominated myocilin (*MYOC*). Linkage analysis led to the identification of the gene encoding this protein as the first glaucoma gene [5]. Since then, this 55 kDa extracellular glycoprotein, has been primarily known by its role in different types of glaucoma [5–8]. This protein belongs to the olfactomedin family of proteins [2] and is secreted into the extracellular space associated with exosomes [9, 10]. It forms extracellular aggregates, which in aqueous humor (AH) range from 120–180 kDa [11] and are in part due to disulfide bonds [12]. Interestingly, *MYOC* mutations cause disease only in the eye by a gain-of-function mechanism [13–17]. Myocilin consists of three independently folded functional domains: i) the N-terminal domain, which contains two coiled coils and one leucine zipper motif, being involved in myocilin self-aggregation [11]; ii) the central linker domain, where calpain II cuts the polypeptide chain, splitting the N- and C-terminal domains [18, 19], and iii) the olfactomedin-like globular domain, which folds as a β-propeller [20] and carry most glaucoma-associated variants [2]. Recently, it has been reported that myocilin is a Y-shaped dimer-of-dimers in which the N-terminal coiled-coil region forms a tetrameric stem linked by disulfide bonds, and the leucine-zipper forms two dimeric arms, connected to two pairs of monomeric OLF domains by a linker region [21]. The biological function of the proteolytic cleavage in the linker domain is not completely understood, but it has been reported to regulate different molecular interactions of recombinant myocilin [22, 23], and it is known to be affected *in vitro* by the extracellular bicarbonate concentration [24]. Fragments of this protein, mainly derived from the C-terminal half of the protein, have also been identified in different glaucoma-related tissues and biological fluids such as the ciliary body, AH [18] and trabecular meshwork [25], indicating that the specific proteolytic cleavage of this protein also occurs *in vivo*. Myocilin activates the Wnt pathway suggesting that this phenomenon might mediate its biological function [26, 27]. Due to its role in glaucoma, myocilin expression has primarily been studied in ocular tissues involved in this disease and its presence in the trabecular meshwork, ciliary body and AH has been well established [2, 18, 25, 28, 29]. Herein, we have explored the localization and biological role of this protein in some non-ocular tissues. We have found that it is present in plasma, some lymphoid tissues and leukocytes and our data also indicate that it reduces the adhesion of human circulating leukocytes to cultured endothelial cell monolayers, providing new insights into the biological properties of myocilin.

## Results

### Expression of myocilin in non-ocular tissues

**Fig 1:**
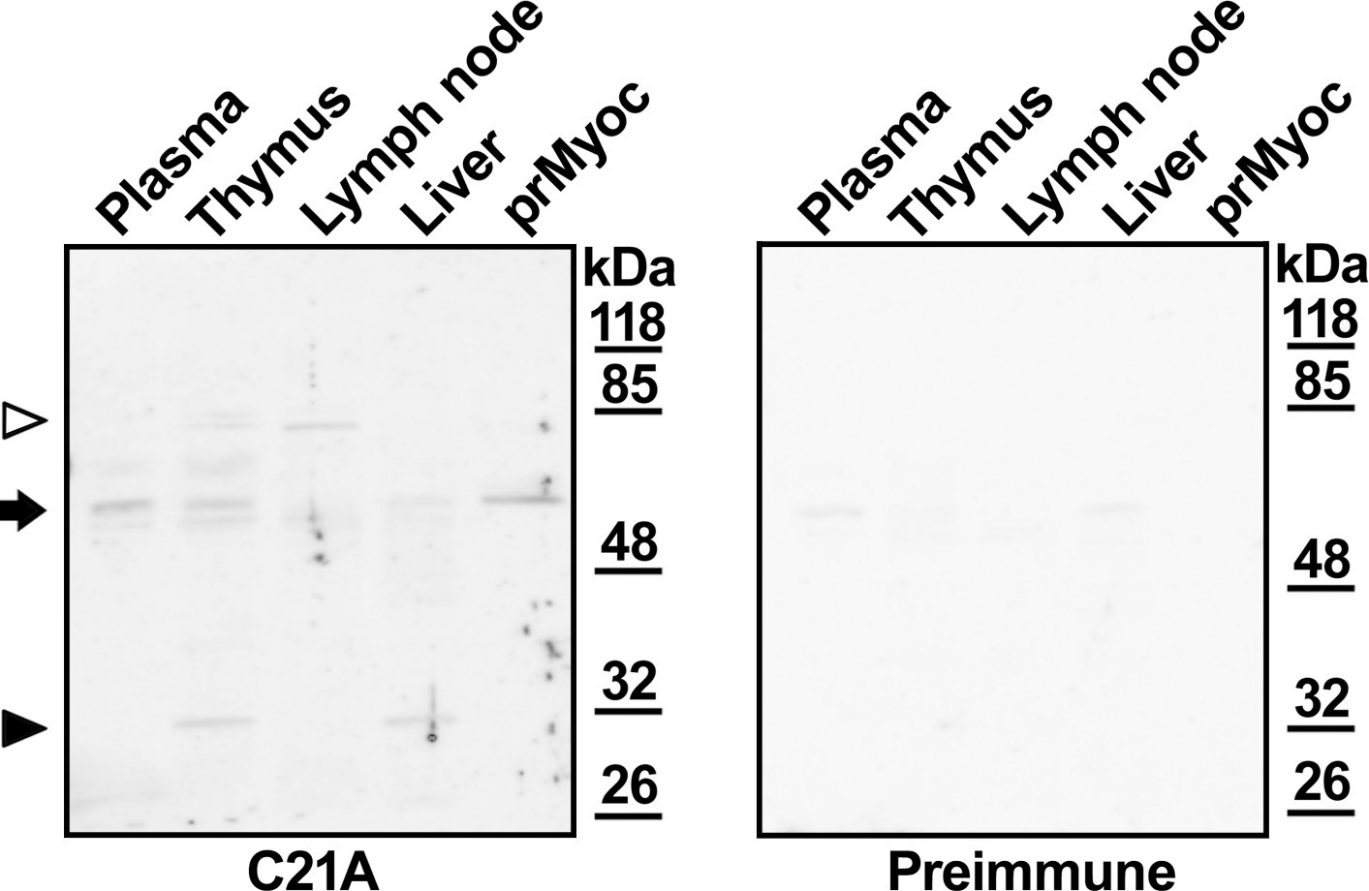
Analysis of myocilin expression in human tissues by Western immunoblot. An aliquot of human plasma and lysates of the different tissues (20 μg total protein), were analyzed by SDS-PAGE. HPLC purified recombinant human myocilin (0.5 μg) was used as a positive control (prMyoc). Myocilin was detected by Western immunoblotting using a chicken IgY polyclonal antibody (C21A). As a negative control a replica of the membrane was analyzed in parallel with the preimmune antibody. Exposure time: 30 s. The Ponceau stained membranes are presented in S2 Fig.

Myocilin was immunodetected by Western blot in different non-ocular organs and tissues. The samples included blood plasma, liver and two representative lymphoid organs (thymus and lymph node). Recombinant myocilin produced in HEK-293T cells was used as a positive control. Myocilin was detected using a chicken IgY polyclonal antibody (C21A), directed against a previously described peptide located in the C-terminal region of the protein [28]. This antibody revealed a characteristic 55 kDa band in all samples (Fig 1, arrow, upper panel), except in lymph node. Interestingly, the C21A antibody recognized specific bands close to the 32 kDa marker in thymus and liver (Fig 1, arrowhead), indicating that they correspond to a C-terminal myocilin fragment. Two 80–85 kDa myocilin aggregates were also detected in thymus and lymph node (Fig 1, white arrowhead, upper panel). As expected, the antibody recognized HPLC purified recombinant myocilin (Fig 1, prMyoc), and these bands were clearly more intense than the weak signals obtained with the preimmune antibody in plasma and liver. To confirm these results myocilin immunodetection was repeated using a commercial purified monoclonal anti-myocilin antibody, which has been described to recognize the N-terminal leucine zipper region of the protein [30], and a highly cross-adsorbed anti-mouse IgG F(ab')2 fragment as secondary antibody. A main band of approximately 55 kDa was detected in all samples (S1 Fig, arrowhead) and specific low molecular weight signals, clearly below the 32 kDa marker, were also observed in lymph node and thymus (S1 Fig, arrow). These signals may correspond to the N-terminal fragment of myocilin, which is predicted to have lower molecular weight than the C-terminal fragment [18]. High concentration of this commercial antibody (1:50) and long exposure time (60 min) were required to detect myocilin, resulting in strong background. These data indicate that the sensitivity of the commercial anti-myocilin antibody is lower than that of the antibody used in this study and support the specificity and sensitivity of the anti-myocilin IgY antibody developed in this work.

**Fig 2:**
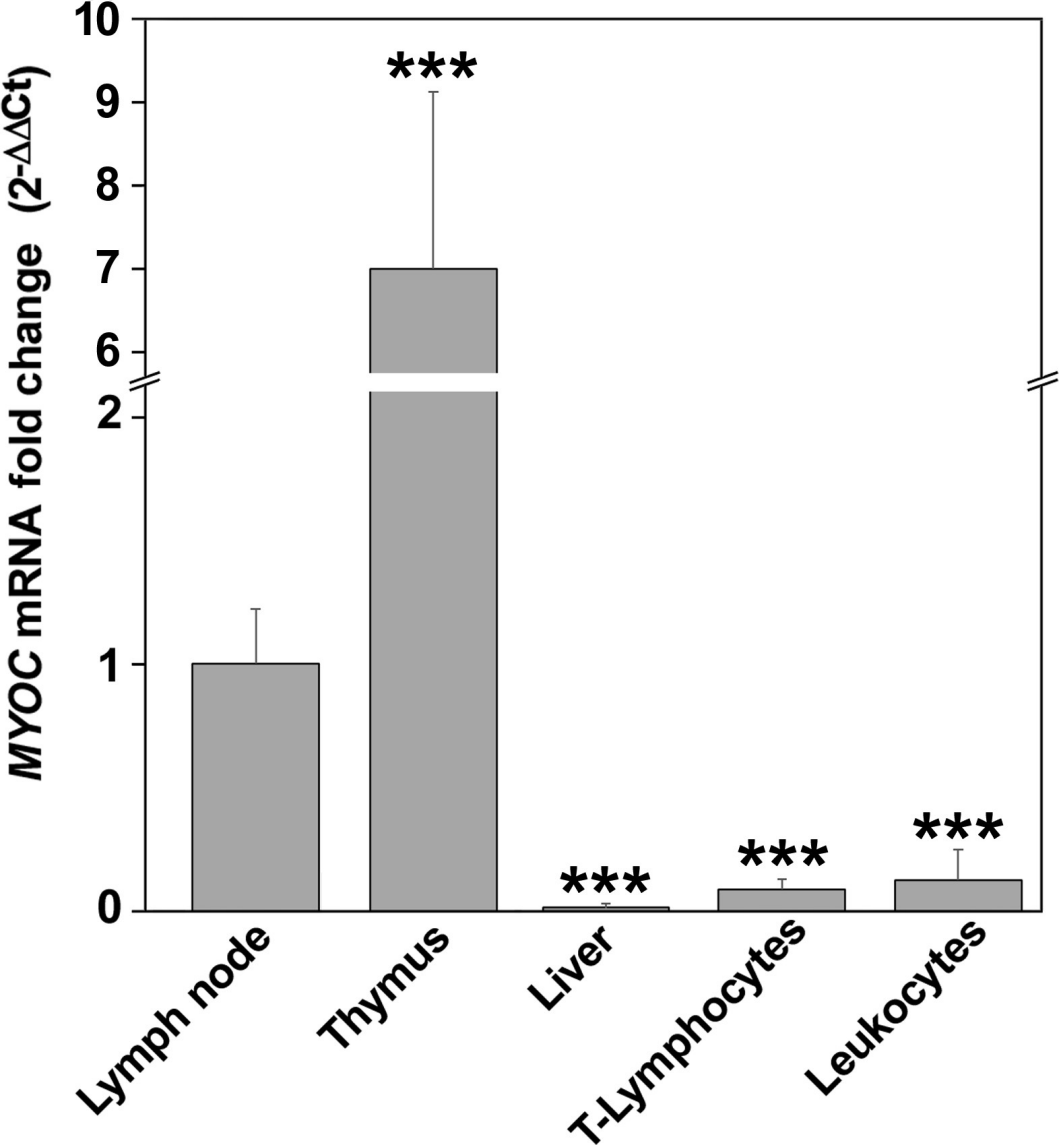
Analysis of myocilin expression in human lymphoid tissues and leukocytes by RT-qPCR. cDNAs (100 ng) were used to amplify myocilin mRNA as indicated in Materials and Methods. The amount of mRNA detected in lymph node was used as a reference to calculate the fold-change of myocilin mRNA. Values are expressed as mean ± SEM of at least three independent experiments carried out in triplicate. Asterisks indicate statistical significance as compared to lymph node: p <0.01 (**); p<0.001 (***); Statistical significance was calculated by Student’s t-test.

*MYOC* expression in liver, lymphoid tissues and lymphocytes was further analyzed by SYBR green real-time quantitative-PCR (qPCR). The highest expression relative to lymph node was observed in thymus (6.9-fold increase) (Fig 2). *MYOC* mRNA was also detected at lower levels in T-lymphocytes and circulating leukocytes (0.12- and 0.15-fold change, respectively). Interestingly, and in accordance with our previous studies, *MYOC* mRNA was absent in liver [2], indicating that although the protein is present in this organ, the gene is not expressed.

### Identification of myocilin as a blood plasma protein

**Fig 3:**
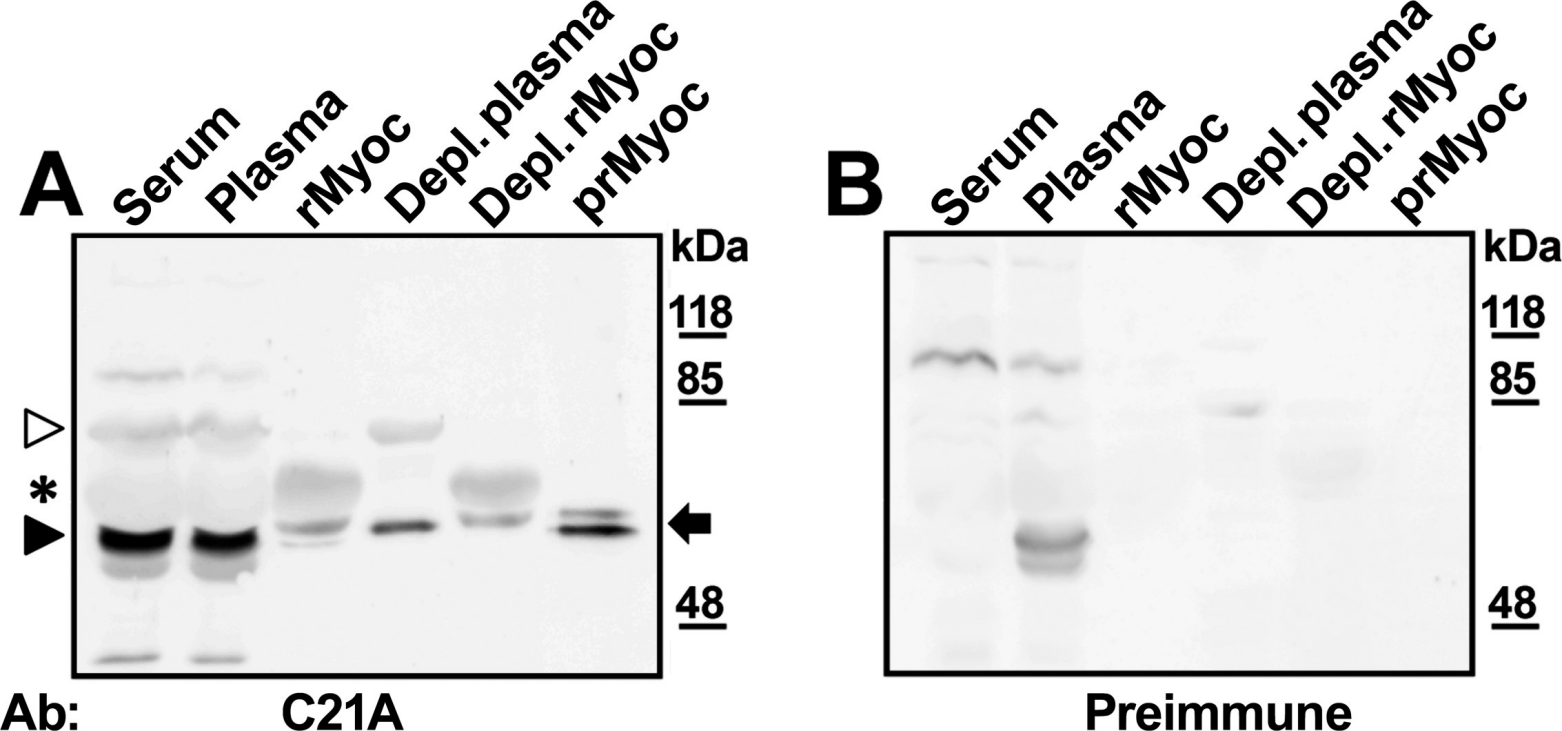
Immunodetection of myocilin in human blood serum and plasma by Western blotting. Samples of human serum and plasma (30 μg total protein each), and albumin and IgG depleted human plasma (40 μg total protein) were included in the analysis. Positive controls consisted of culture medium containing recombinant myocilin (rMyoc) expressed in HEK-293T cells and one microgram of HPLC-purified recombinant myocilin (prMyoc). As a control of depletion, a sample of culture medium containing recombinant myocilin was treated in parallel (Depl. rMyoc). Electrophoresis was performed on an 8% polyacrylamide gel and the anti-myocilin C21A polyclonal antibody was used. As a negative immunodetection control, samples were analyzed in parallel with the corresponding preimmune antibody (Preimmune). Exposure time: 1 min. The asterisk indicates the position of non-specific albumin signals. The white arrowhead indicates the position of a myocilin aggregate. The black arrowhead shows the altered electrophoretic mobility of myocilin in serum and plasma produced by albumin and the black arrow indicate the position of the recombinant myocilin monomer in the different samples. Full-length blots and Ponceau S stained membranes are presented in S4 Fig.

**Fig 4:**
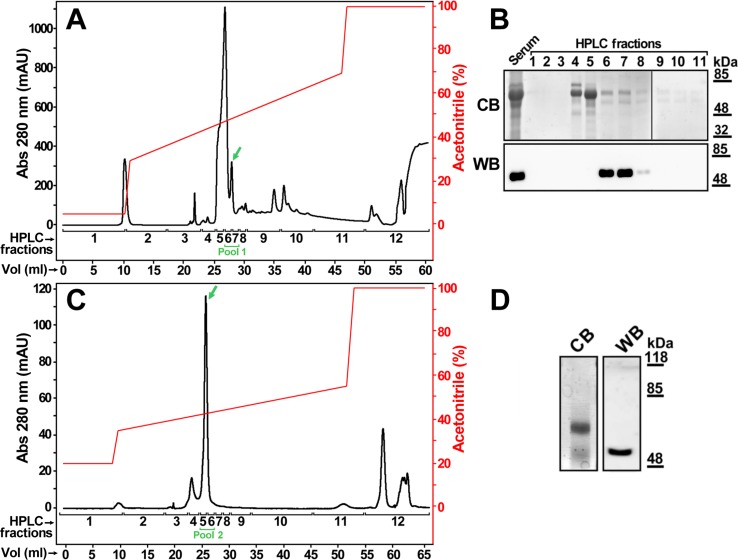
Isolation of myocilin from human blood serum by reverse-phase HPLC. **(A)** A sample of human serum was processed as indicated in materials and methods. The sample was eluted using the acetonitrile gradient shown by the red line. The collected fractions (1–12) are indicated above the abscissa axis. **(B)** Aliquots of the chromatographic fractions were analyzed by SDS-PAGE and the eluted proteins were detected by Coomassie blue staining (CB). Fractions containing myocilin were identified by Western blot (WB) using an anti-myocilin C21A polyclonal antibody (WB). The full-length blot is presented in S5 Fig. **(C)** Pool 1 from panel A was refractionated in the same chromatographic system using the indicated acetonitrile gradient. **(D)** Fractions 5 and 6 from panel C were analyzed by SDS-PAGE and proteins were also detected by Coomassie blue staining and Western Blot as indicated in B. To maximize band separation the electrophoresis was run until the 48 kDa marker reached the bottom of the gel. The full-length polyacrylamide gel is shown in S6 Fig. Green arrows in (A) and (C) indicate the elution peak corresponding to myocilin. Fractions 6 and 7 from (A) and fractions 5 and 6 from (C) were mixed to form Pool 1 and Pool 2, respectively.

In order to confirm the interesting finding of myocilin in human blood, samples of plasma, serum, and albumin-depleted plasma were analyzed by Western immunoblotting. This analysis revealed a major broad 50 kDa-band in serum and plasma, and a 85 kDa-signal in the three samples (Fig 3A, black and white arrowheads, respectively). The abundance of albumin (Fig 3A, asterisk) increased the electrophoretic mobility of myocilin, both in serum and plasma. In fact, albumin-depleted plasma, in addition to the 85 kDa band (Fig 3A, white arrowhead), showed one band of approximately 55 kDa (Fig 3A, black arrow). Comparison of these signals and those obtained with the preimmune C21A antibody (Fig 3B) indicated that some 55 kDa overlapping signals were nonspecific and correspond to unidentified plasma proteins that were removed by coagulation. The electrophoretic mobility of plasma myocilin after albumin depletion was similar to that of purified recombinant protein and to that of the recombinant protein detected in the conditioned culture medium after bovine albumin depletion (positive controls). Although bovine albumin was removed less efficiently than human albumin by the immunoaffinity kit, these results provide further support for the presence of myocilin in human blood plasma. Overall, this analysis showed that myocilin is present in albumin-depleted plasma as a 55 kDa-monomer and an 85 kDa-complex. To confirm the presence of myocilin in human blood serum we isolated the protein by reverse-phase HPLC (Fig 4A). SDS-PAGE and Western immunoblot of the chromatographic fractions revealed the presence of myocilin in fractions six to eight, which were composed of various proteins according to electrophoretic analysis with Coomassie Blue staining (Fig 4B). To increase the purity of myocilin, these three fractions were pooled and rechromatographed using a lower slope acetonitrile gradient (Fig 4C). Fractions corresponding to the main peak (five and six) were mixed and analyzed again by SDS-PAGE. Combined Coomassie Blue staining and Western blotting immunodetection showed a partially purified myocilin preparation (Fig 4D). The lower Coomassie blue stained band was cut from the gel and subjected to in-gel trypsin digestion and MALDI-TOF peptide mass fingerprint. This analysis led to the identification of 39 myocilin peptides (Table 1) out of 295 (13%), which covered 64% of the myocilin amino acid sequence (S3 Fig). Altogether these results demonstrated the presence of myocilin in human blood serum.

**Table 1:** Myocilin peptides identified by MALDI-TOF analysis after in-gel trypsin digestion of pool 2 (Fig 4C and 4D) resulting from reverse-phase HPLC fractionation of human blood serum.

m/z observed	m/z theoretical	Δm/z (%)	Number of oxidized amino acids	^a^Amino acid residues	Peptide sequence	Missed trypsin target sites	Cumulative sequence coverage (%)
**2976.114**	**2978.4395**	**0.08**	**1**	**8–33**	^b^**(R)CCSFGPEMPAVQLLLLACLVWDVGAR(T)**	**0**	**22.2**
**3175.493**	**3177.6498**	**0.07**	**1**	**92–119**	**(R)LSSLESLLHQLTLDQAARPQETQEGLQR(E)**		
**1894.914**	**1893.9593**	**0.05**	**0**	**201–216**	**(R)EVSTWNLDTLAFQELK(S)**		
**1883.802**	**1882.897**	**0.05**	**0**	**273–287**	**(R)DPKPTYPYTQETTWR(I)**		
**1460.744**	**1459.6774**	**0.07**	**0**	**473–484**	**(K)YSSMIDYNPLEK(K)**		
**1475.859**	**1475.6723**	**0.01**	**1**	**473–484**	**(K)YSSMIDYNPLEK(K)**		
**1857.929**	**1858.9044**	**0.05**	**1**	**486–500**	**(K)LFAWDNLNMVTYDIK(L)**		
**1275.778**	**1275.6288**	**0.01**	**0**	**127–136**	**(R)ERDQLETQTR(E)**	**1**	**45.8**
**2278.954**	**2280.1466**	**0.05**	**0**	**129–147**	**(R)DQLETQTRELETAYSNLLR(D)**		
**1289.813**	**1289.6015**	**0.02**	**0**	**183–193**	**(R)GQCPQTRDTAR(A)**		
**1142.722**	**1142.5913**	**0.01**	**1**	**190–200**	**(R)DTARAVPPGSR(E)**		
**2574.492**	**2574.3198**	**0.01**	**1**	**194–216**	**(R)AVPPGSREVSTWNLDTLAFQELK(S)**		
**2590.694**	**2590.3148**	**0.01**	**2**	**194–216**	**(R)AVPPGSREVSTWNLDTLAFQELK(S)**		
**1442.869**	**1442.8213**	**0.00**	**0**	**217–229**	**(K)SELTEVPASRILK(E)**		
**1458.693**	**1458.8162**	**0.01**	**1**	**217–229**	**(K)SELTEVPASRILK(E)**		
**1629.831**	**1628.8101**	**0.06**	**1**	**259–272**	**(R)TAETITGKYGVWMR(D)**		
**2707.216**	**2707.2609**	**0.00**	**2**	**267–287**	**(K)YGVWMRDPKPTYPYTQETTWR(I)**		
**2721.768**	**2723.2559**	**0.05**	**3**	**267–287**	**(K)YGVWMRDPKPTYPYTQETTWR(I)**		
**2872.215**	**2871.3795**	**0.03**	**2**	**273–296**	**(R)DPKPTYPYTQETTWRIDTVGTDVR(Q)**		
**1566.916**	**1565.8533**	**0.07**	**0**	**343–355**	**(R)TVIRYELNTETVK(A)**		
**2785.545**	**2783.4574**	**0.07**	**1**	**399–422**	**(K)GAIVLSKLNPENLELEQTWETNIR(K)**		
**1751.797**	**1750.8356**	**0.05**	**0**	**471–484**	**(R)YKYSSMIDYNPLEK(K)**		
**1586.577**	**1587.7723**	**0.08**	**0**	**473–485**	**(K)YSSMIDYNPLEKK(L)**		
**2171.434**	**2171.1205**	**0.01**	**0**	**486–503**	**(K)LFAWDNLNMVTYDIKLSK(M)**		
**1444.656**	**1444.7615**	**0.01**	**0**	**34–46**	**(R)TAQLRKANDQSGR(C)**	**2**	**64.3**
**1691.667**	**1690.8719**	**0.05**	**0**	**77–91**	**(R)DSSTQRLDLEATKAR(L)**		
**2367.418**	**2366.2086**	**0.05**	**0**	**137–156**	**(R)ELETAYSNLLRDKSVLEEEK(K)**		
**2217.171**	**2216.1014**	**0.05**	**0**	**161–179**	**(R)QENENLARRLESSSQEVAR(L)**		
**1530.596**	**1530.8347**	**0.02**	**0**	**169–181**	**(R)RLESSSQEVARLR(R)**		
**1445.778**	**1445.7026**	**0.01**	**0**	**182–193**	**(R)RGQCPQTRDTAR(A)**		
**1461.753**	**1461.6975**	**0.00**	**1**	**182–193**	**(R)RGQCPQTRDTAR(A)**		
**3331.425**	**3333.7577**	**0.07**	**1**	**201–229**	**(R)EVSTWNLDTLAFQELKSELTEVPASRILK(E)**		
**2332.544**	**2332.2507**	**0.01**	**0**	**217–237**	**(K)SELTEVPASRILKESPSGYLR(S)**		
**2348.439**	**2348.2456**	**0.01**	**1**	**217–237**	**(K)SELTEVPASRILKESPSGYLR(S)**		
**2363.635**	**2364.2405**	**0.03**	**2**	**217–237**	**(K)SELTEVPASRILKESPSGYLR(S)**		
**3506.444**	**3507.7424**	**0.04**	**2**	**227–258**	**(R)ILKESPSGYLRSGEGDTGCGELVWVGEPLTLR(T)**		
**1894.914**	**1894.028**	**0.05**	**0**	**343–358**	**(R)TVIRYELNTETVKAEK(E)**		
**2035.489**	**2036.9746**	**0.07**	**1**	**469–484**	**(K)NRYKYSSMIDYNPLEK(K)**		
**1894.914**	**1894.9255**	**0.00**	**1**	**471–485**	**(R)YKYSSMIDYNPLEKK(L)**		

^a^The numbers indicate the position of the amino acids in the polypeptide chain of myocilin.

^b^The letters between parentheses indicate the amino acid residues flanking the different trypsin cleavage sites.

**Fig 5:**
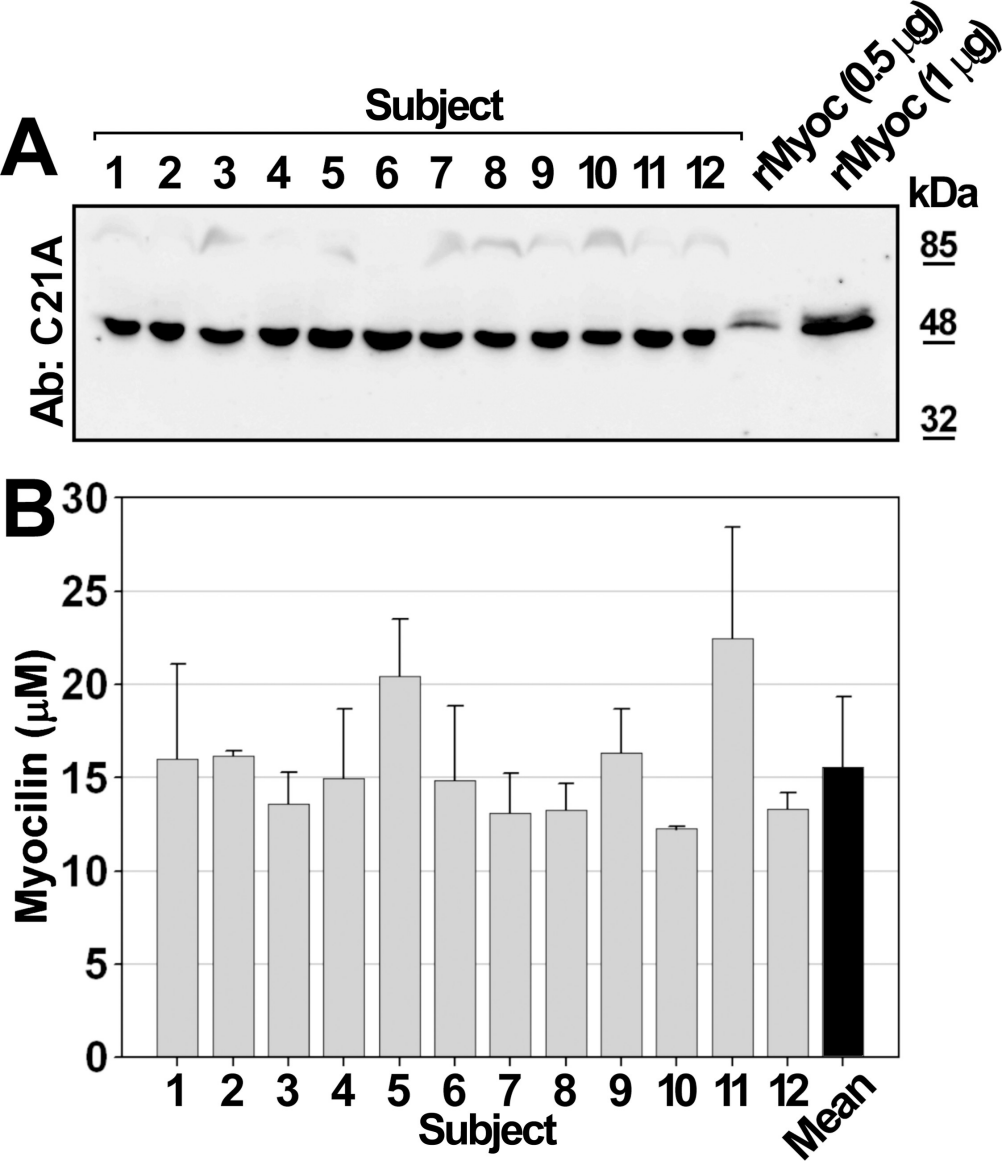
Estimation of myocilin concentration in human blood serum by Western immunoblot. **(A)** Human blood serum aliquots (30 μg total protein) from control subjects (1–12) were analyzed by Western immunoblot using the chicken polyclonal anti-myocilin C21A antibody. Samples containing 0.5 μg and 1 μg of HPLC purified recombinant human myocilin were used as a reference for densitometry. Each sample was analyzed in triplicate. The image shows a representative Western blot. The full-length membrane is shown in S5 Fig. **(B)** Estimated myocilin concentration in blood serum samples from 12 human donors. Values are expressed as mean ± SEM of triplicates from two independent analyses. rMyoc: recombinant myocilin.

Next, we estimated by Western immunoblot and densitometry the concentration of myocilin in human blood serum samples obtained from 12 control individuals (Fig 5A). All serum samples showed a main 55 kDa-myocilin band, corresponding to the monomer, as well as low-abundance 90 kDa-complexes. Gross densitometric estimation showed a mean concentration of serum myocilin of 15.5± 3 μM (approximately 0.85±0.16 mg/ml), with individual values varying from 12 μM (subject 1) to 23 μM (subject 5) (Fig 5B).

### Effect of myocilin on adhesion of circulating human leukocytes to endothelial cells

**Fig 6:**
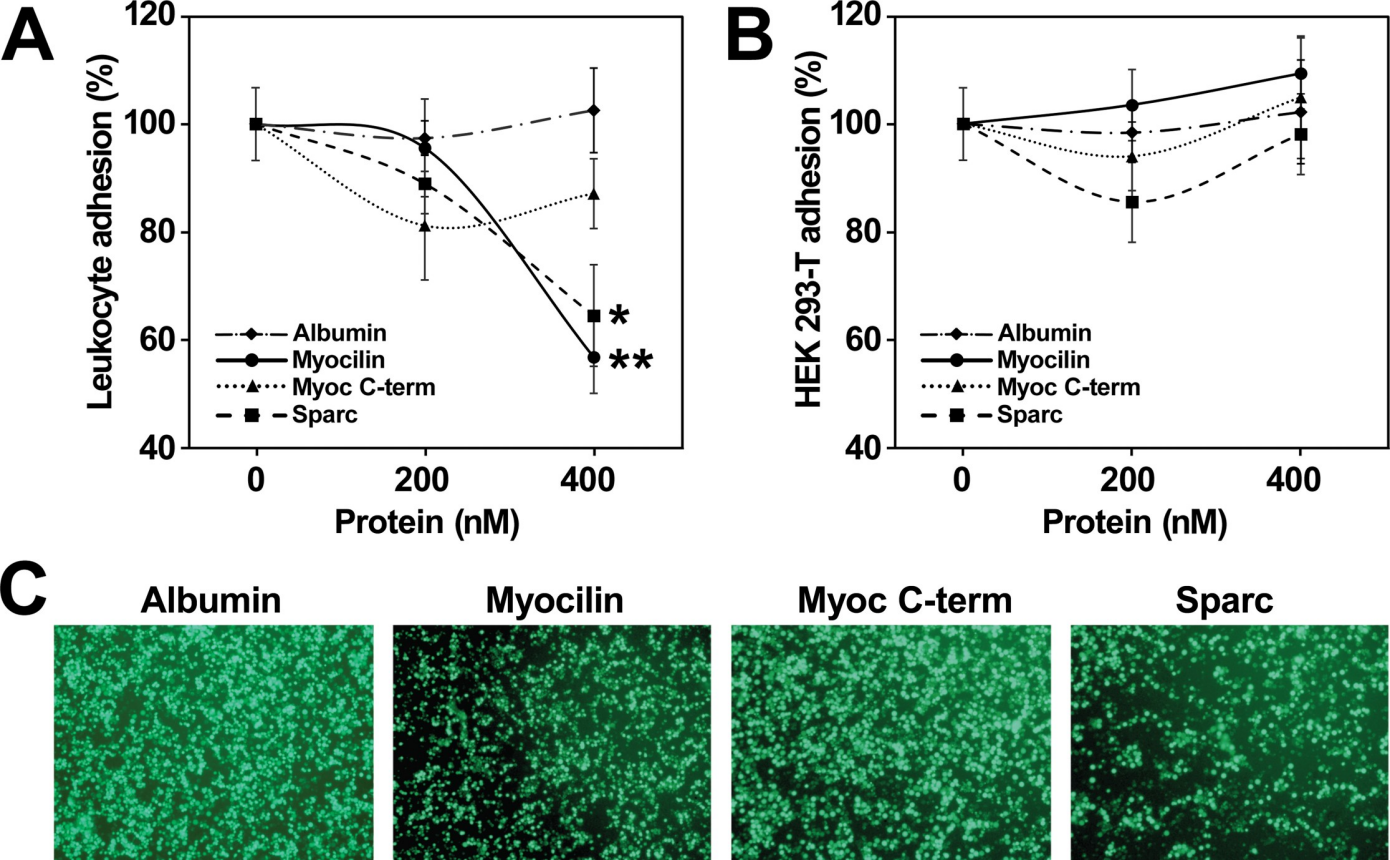
Effect of recombinant myocilin on human leukocyte adhesion to endothelial cell monolayers (HUVEC). **(A)** Calcein-labelled human leukocytes were isolated and incubated with different concentrations of the indicated purified recombinant proteins as described in Materials and Methods. Then, leukocytes were added to TNFα activated HUVEC cell monolayers. Adhered cells were quantified by fluorescence). Purified bovine serum albumin (Albumin) was used as a negative control of cell adhesion. Cell adhesion was expressed as a percent of adhered cells in the absence of assayed proteins. **(B)** As a control of cell specificity, the assay was performed in parallel with HEK-239-T cells. Percentage of adhesion values are expressed as mean ± SEM of at least two independent experiments carried out in triplicate. **(C)** Fluorescence microscopy of leukocytes adhered to HUVEC cells. The cells were treated as indicated in A. Ten random fields were observed on a Nikon Eclipse Ti fluorescence microscope and representative photographs are shown. Magnification: 40X. Statistical differences were calculated using the one-way ANOVA test. p <0.05 (*); p <0.01 (**).

The presence of myocilin in plasma and lymphoid tissues, along with its proposed effect on cell adhesion [31], prompted us to evaluate its possible effect on leukocyte adhesion to endothelial cells in culture. To this end, isolated circulating human leukocytes were treated as described in Materials and Methods. We observed that myocilin produced a significant and dose-dependent reduction of leukocyte adhesion to HUVEC monolayers (Fig 6A), similar to that of the control antiadhesive protein SPARC [32]. The myocilin C-terminal fragment also showed a significant antiadhesive effect, although it was less intense than that of the full-length protein and was saturated at a concentration of 200 nM (Fig 6A). In a parallel assay we evaluated the adhesion of HEK-293T cells to HUVEC cells and although an increased adhesion tendency was observed with myocilin, the differences were not statistically significant (Fig 6B). These data indicate the specificity of the detected antiadhesive effect and that it may depend on the cell type. The reduction of adhered leukocytes to HUVEC monolayers by the different molecules at a concentration of 400 nM was confirmed by microscopic examination of the samples (Fig 6C).

## Discussion

Up to date, most myocilin research have focused on ocular tissues due to its role in glaucoma. Studies of the expression of myocilin at the protein level have been hampered by limited specificity of available antibodies. In this study, we have explored the presence and biological function of myocilin in non-ocular tissues, using a chicken IgY antibody raised against a previously described myocilin epitope located in the C-terminal region of the protein [28]. The phylogenetic distance between chicken and human proteins allowed us to improve sensitivity and to reduce background noise in myocilin detection, using IgY antibodies [33]. This antibody detected the myocilin monomer (55 kDa) in human blood plasma, thymus and liver. In accordance with this result, our previous studies using a rabbit anti-myocilin antibody against the same epitope also detected a 55 kDa band in several human ocular tissues, including iris, ciliary body and trabecular meshwork [28]. An 80–85 kDa myocilin aggregate was also detected in lymph node as will be explained later. qPCR confirmed the expression of *MYOC* in these two lymphoid organs (lymph node and thymus) as well as in circulating leukocytes and T-lymphocytes, but not in liver. In accordance with these results two previous studies detected *MYOC* mRNA by Northern blot in thymus [34, 35]. However, these reports did not identify *MYOC* mRNA in leukocytes, probably due to the low sensitivity of Northern blot compared with qPCR. Likewise, a previous study also detected *MYOC* transcripts in ficoll-isolated circulating human leukocytes, which are enriched in lymphocytes [36]. The identification of 30 kDa C-terminal fragments of myocilin in thymus and lymph node suggests the existence of a tissue/organ-specific myocilin proteolytic processing. These results also support a functional role for this posttranslational processing, which was initially demonstrated in the recombinant protein [18, 19]. According to our previous studies this proteolytic processing might regulate myocilin aggregation [22], but additional studies are required to clarify this point. Myocilin fragments present in liver might have a different functional meaning because this organ does not express detectable myocilin. Therefore, it can be speculated that the liver might be involved in plasma myocilin turnover. Other molecular forms of myocilin are indicated by the presence of 80–85 kDa myocilin bands observed in thymus, lymph node and also in plasma, which might correspond to heterocomplexes of myocilin and unidentified proteins orto complexes of full-length myocilin and myocilin fragments. In line with these ideas, it is known that myocilin forms extracellular multimers [11], which are maintained by posttranslational disulfide bond formation [12, 21, 22] and non-covalent interactions between the leucine zipper motifs [11, 22]. Detailed medical records were not available from donors of the different commercial tissue samples and, although unlikely, we cannot completely rule out any that myocilin detected in these samples is affected by unreported diseases. Further investigations are required to clarify these issues.

The presence of myocilin in blood serum was demonstrated by peptide mass fingerprint analysis of the protein isolated by reverse-phase chromatography. In addition, the concentration of myocilin, roughly estimated from a group of normal human blood serum samples (15.5 μM or 0.8 mg/ml), indicated that it is a medium-abundance serum protein accounting approximately for one per cent of the total serum protein (considering a total protein concentration in human serum of 65–78 mg/ml). These results show that myocilin can be included into the group of plasma proteins that are also expressed in the ciliary body and secreted to the AH (*e*.*g*., complement component C4, alpha-2 macroglobulin, selenoprotein P and apolipoprotein D) [1]. Because the liver does not express myocilin, immune cells and organs could be a source for this protein present in plasma. Myocilin has also been identified in the AH at a concentration of approximately 0.2–0.6 ng/μl [37]. The concentration determined in this study for myocilin in human serum is several orders of magnitude higher than the concentration reported in AH, which is not surprising since the protein content of human AH is extremely low, containing between 120 and 500 ng/μl of total protein [38] (*i*.*e*., almost 200–500 times less than plasma). In accordance with our results two previous studies carried out either to characterize the human plasma proteome [39] or to verify plasma biomarkers for diabetic retinopathy [40], identified three myocilin peptides in plasma.

The presence of myocilin in plasma and lymphoid tissues, which are involved in leukocyte trafficking, and the reported role of this protein on cell adhesion [41, 42], led us to hypothesize the possible role of myocilin on leukocyte adhesion. To the best of our knowledge, our data evidence for the first time that recombinant myocilin reduces adhesion of peripheral human leukocytes to monolayers of cultured endothelial cells (HUVEC) and that this feature is associated, at least in part, with the olfactomedin-containing C-terminal part of the protein. According to this result, it has also been described an anti-adhesive myocilin activity on trabecular meshwork cells that may be mediated by a reduction of focal adhesions and stress fibers actin [41, 42]. The elevated purity of the HPLC-purified recombinant proteins used in this study, assessed by SDS-PAGE and silver nitrate staining [23], and the differences in antiadhesive properties shown by full-length myocilin and its C-terminal fragment support that myocilin is the active component of the preparations used to evaluate leukocyte adhesion. The effect of myocilin on cell adhesion may differ depending on the cell type and/or experimental settings since it is also known that immobilized recombinant myocilin promotes substrate adhesion of podocytes and mesangial cells [31]. Moreover, and in line with our finding, two matricellular and myocilin-interacting proteins SPARC [32] and hevin [43] inhibit endothelial cell adhesion to some substrates. Myocilin exhibits several features found among SPARC proteins, including counter-adhesive properties [43], which is a key functional signature of matricellular proteins [44]. These are a group of proteins involved in cell-to-cell interactions and in the biochemical interplay between cells and extracellular environment [45]. Leukocyte adhesion to endothelial cells is principally mediated by selectins and integrins [46]. Therefore, we can hypothesize that plasma myocilin disrupts or interfere the interaction of these proteins leading to reduced adhesion of leukocytes to endothelial cells, although further work is required to evaluate this hypothesis. Endoproteolytic regulatory cleavage and expression in lymphoid tissues are additional characteristics present in myocilin, SPARC and hevin [45]. Altogether, these data also suggest a role for myocilin as a blood matricellular protein. The adhesion of circulating leukocytes to activated endothelial cells is a critical step in inflammation and in the process of leukocyte extravasation [47], in which hevin has been proposed to play a role [43]. Therefore, our data, together with the presence of myocilin in plasma and its expression in lymphoid tissues, suggest that myocilin also might participate in the initial steps of inflammation, although this is a speculative idea that needs further investigation. Finally, and in accordance with this hypothesis, it is well known that the expression of myocilin is induced by glucocorticoids [4], which are involved in inflammation.

## Conclusions

In summary, our results demonstrate that human myocilin is present in blood plasma, as well as in some lymphoid tissues and leukocytes. In addition, our study indicates a role of myocilin on leukocyte adhesion to cultured human endothelial cell monolayers and provide insights for new biological properties of myocilin and for its putative role as a blood matricellular protein.

## Materials and methods

### Ethics statment

Human blood samples from normal volunteers were collected with informed and written consent in compliance with the Declaration of Helsinki ethical principles, and their use was approved by the Institutional Ethics Committee of “Complejo Hospitalario Universitario de Albacete”, Spain.

### Chicken polyclonal antibody production

An affinity-purified chicken anti-myocilin antibody raised against a previously described synthetic peptide was obtained by Immunostep. The peptide corresponded to amino acids 468–488 (C21A: CNRYKYSSMIDYNPLEKKLFA) [19, 28] of the human protein of the human protein and contained an extra Cys residue in its N-terminal end to facilitate conjugation to Keyhole Limpet Hemocyanin peptide. After immunization, IgY was purified from the egg yolk using the Pierce Chicken IgY Purification Kit (ThermoFisher Scientific).

### Western blotting

Protein extracts from the following adult normal human tissues were used for western blotting analyses: liver and thymus (BioChain) and lymph node (Clontech). Age and sex of the donors of tissues were as follows: liver, 87-years-old female; thymus, 36-years-old male; lymph node, pooled from 59 male/females, 19-68-years-old. The reported causes of death were trauma or sudden death. A venous blood sample from one healthy 55-years-old male volunteer member of the research group was used for HPLC fractionation. Recombinant human myocilin used as a positive control was produced in HEK-293T cells and purified by Ni-chelating HPLC as described elsewhere [22]. Albumin and IgG were removed from human plasma and conditioned culture medium samples using the ProteoPrep Immunoaffinity Albumin and IgG Depletion Kit (Protea Biosciences) following the manufacturer’s instructions. Aliquots of control serum samples selected from patients undergoing cataract surgery from a previous proteomic study [48] were used to estimate myocilin concentration. The protein content of samples was determined by the Bicinchoninic Acid Protein Assay Kit (Thermo Scientific), following the manufacturer’s recommendations. Prior to electrophoresis the samples were boiled for 5 min in standard electrophoresis loading buffer containing β-mercaptoethanol, then they were usually subjected to 10% polyacrylamide gel electrophoresis in the presence of SDS [49], using the Mini-PROTEAN III gel electrophoresis system (BioRad). Gels were subsequently transferred onto Hybond ECL nitrocellulose membranes (Amersham) for immunodetection. Ponceau S (Panreac) staining of blots prior to antibody incubation was performed to confirm the integrity of samples and that equal amounts of sample were analyzed [50]. The C21A was used as primary antibody (1:500 dilution) and an anti-chicken IgY horse-radish peroxidase-conjugated (1:1000 dilution) was employed as secondary antibody (Santa Cruz). To assess the specificity of the antibody replicas of the samples were analyzed in parallel with the preimmune antibody (1:500 dilution). To confirm the specificity of the C21A antibody a commercial purified monoclonal anti-myocilin antibody (MAB3446, R&D Systems, MN, USA) was used at a 1:50 dilution. This monoclonal antibody has been described to recognize the N-terminal leucine zipper region of the protein [30]. A highly cross-adsorbed anti-mouse IgG F(ab')2 fragment (SAB3701015, Sigma-Aldrich) at a 1:500 dilution was employed as secondary antibody. Chemiluminescence analysis was performed with Supersignal Dura Western Blot reagents (Thermo Scientific) using the LAS3000-mini (Fujifilm, Tokyo, Japan) detector. Densitometry for protein band quantification was performed using Quantity One 4.1 analysis software (BioRad) in at least two independent experiments performed in triplicate.

### Analysis of *MYOC* gene expression by real-time quantitative PCR (RT-qPCR)

Commercial cDNA samples of human tissues were used for RT-qPCR (Human BioBank cDNA for real-time PCR, Pimer Design Ltd). Expression of the different mRNAs relative to GAPDH mRNA was determined using the 2^−ΔΔCt^ method [51] and the primer pair 5’-AGAAGGCTGGGGCTCATTTG-3’ and 5’-AGGGGCCATCCACAGTCTTC-3’. The following primers were employed to amplify myocilin cDNA: 5’-AGGTTGGAAAGCAGCAGCCAGG-3’ and 5’-TGCTGTTCTCAGCGTGAGAGG-3’. For PCR analysis, 1 μl of cDNA was used as a template in a reaction volume of 10 μl containing 5μl of Power SYBR Green PCR Master Mix (Thermo Fisher Scientific) and 200 nM of each primer. Nuclease-free water was added up to 10 μl. Thermocycling included an initial denaturation step at 95º C for 10 min, followed by 40 cycles consisting of 15 s denaturation at 95º C followed by 60 s of annealing and extension at 60ºC. The PCR products and their dissociation curves were detected with an ABI PRISM 7500 Fast real-time PCR system (Life Technologies). Quantitative PCR results from at least three independent experiments carried out in triplicate were used for calculation of mean expression values in each tissue.

### Isolation of serum myocilin by high performance liquid chromatography (HPLC)

Serum samples were obtained from blood without anticoagulant. Once the serum was aliquoted, 100 μl of sample previously acidified with 0.1% (v/v) trifluoroacetic acid (TFA) were used and centrifuged to remove the pellet. The serum was injected into a reverse-phase column (Discovery BIO Wide Pore C5, Supelco) coupled to an Akta Purifier (Amersham Biosciences) chromatograph. Prior to sample loading the column was equilibrated with H_2_O containing 0.1% TFA at a flow rate of 1 ml/min. The sample was eluted with linear gradients of acetonitrile containing 0.1% (v/v) TFA, as indicated in the corresponding figures. Fractions were manually collected, and their purity was assessed by SDS-PAGE with Coomassie blue staining. The presence of myocilin in the different fractions was analyzed by Western immunoblot using the C21A antibody (1:500 dilution). Fractions containing myocilin were pooled and subjected to a second round of purification using a lower slope acetonitrile gradient (38% to 58% acetonitrile in 45 min). The samples containing myocilin were further fractionated by SDS-PAGE and the bands obtained were cut from the gel and analyzed by mass spectrometry.

### Matrix-Assisted laser desorption ionization time-of-flight (MALDI-TOF) peptide mass fingerprint analysis

The mass spectrometry analysis was performed at the Proteomics Service of Madrid Science Park. The bands obtained by SDS-PAGE were excised with a scalpel and subjected to in-gel trypsin digestion [52] and the resulting peptides were identified by mass spectrometry in a MALDI-TOF/TOF 4700 Proteomics Analyzer (Applied Biosystems). External calibration was performed using a mixture of angiotensin II, ACTH/CLIP, bombesin and somatostatin. The prediction of peptides resulting from trypsin digestion of myocilin was performed using the MS-Digest tool (http://prospector.ucsf.edu/prospector/cgi-bin/msform.cgi?form=msdigest). The theoretical mass of these peptides was compared to the experimentally determined mass using the Mascot Search Results online tool (www.matrixscience.com). Finally, the percentage of the myocilin sequence covered by the tryptic peptides identified by mass spectrometry was determined.

### Estimation of the concentration of myocilin in human plasma

Serum samples from control subjects were subjected to SDS-PAGE (10% polyacrylamide) followed by Western immunoblotting using the anti-myocilin antibody C21A (Immunostep) (1:500 dilution), as described earlier. The signals obtained from two known quantities (0.5 and 1.0 μg) of Ni-chelating HPLC-purified recombinant human myocilin [23] were used as a reference for densitometric quantification of myocilin detected by western immunoblotting using the Quantity One 4.6.9. software (Bio-Rad). To identify nonspecific signals an aliquot of each sample was analyzed in parallel using the preimmune antibody (1:500 dilution). Values were calculated from two independent assays carried out in triplicate.

### Assay of leukocyte adhesion to endothelial cells in culture

The human umbilical vein endothelial cells (HUVEC, ECACC 06090720, Sigma-Aldrich) used in the assay were cultured in Complete Endothelial Cell Growth Medium (ECACC 06091509). The human embryonic kidney 293T cell line (HEK-293T), used as a negative control, was obtained from the ATCC (American Type Culture Collection) and was maintained in Dulbecco’s modified Eagle’s medium (DMEM, Lonza) supplemented with 10% fetal bovine serum (FBS). The two cell lines were cultured with antibiotics (Normocin, Invitrogen) at 37°C in a fully humidified 5% CO_2_ atmosphere. To evaluate the effect of myocilin on the adhesion of leukocytes to endothelial cells, HUVEC cells were seeded in black-walled transparent-bottom culture plates (Greiner Bio-One), treated with 0.1% gelatin (Sigma-Aldrich). Confluent cells were treated for 4 h with culture medium containing 10 ng/ml of TNFα (Sigma-Aldrich) and different concentrations of recombinant proteins. Circulating leukocytes were obtained from peripheral venous blood anticoagulated with heparin. The blood (10 ml) was diluted with DPBS (1:1) and carefully placed onto an equivalent volume of Ficoll-Paque PLUS (GE Healthcare Life Sciences). The sample was centrifuged (800xg) at room temperature for 30 min. The leukocyte rich layer was collected and washed twice with Dulbecco's Phosphate Buffer Saline (DPBS, Lonza). Leukocytes activation was performed by incubation with phytohemagglutinin (10 ng/ml) for 20 min in RPMI-1640 complete medium (Lonza) containing 10% FBS. Activated leukocytes (5000000 cells/ml) were incubated at 37° C for 30 min in RPMI-1640 containing 5 μM calcein-AM (AnaSpec). After two RPMI-1640 washes, calcein-labelled leukocytes in the same medium were seeded (500000 cells/well) on HUVEC monolayers, at 37ºC for 2 h, in the presence of two concentrations of recombinant myocilin or SPARC (200 nM and 400 nM). The recombinant proteins were produced in HEK-293T cells and purified from the culture medium by Ni-chelating HPLC as previously described [22, 23]. The purity of the obtained recombinant proteins assessed by SDS-PAGE with silver nitrate staining was at least 80% [23]. As a control of cell specificity, calcein-labelled HEK-293-T cells (500000 cells/well) were added in parallel to HUVEC monolayers. Non-adherent cells were removed by three DPBS washes. The fluorescence of each well was quantified in a XS Gemini plate fluorimeter (Molecular Devices) using excitation and emission wavelengths of 494 nm and 517 nm, respectively. Purified bovine serum albumin (200 nM and 400 nM) was used as a negative control of cell adhesion (Sigma-Aldrich). Cell adhesion was expressed as a percent of adhered cells in the absence of assayed proteins. In addition, photographs of the preparations were obtained using a Nikon Eclipse Ti-U (Nikon) fluorescence microscope, equipped with a Nikon-cooled digital camera DS-Ri1 (Nikon).

### Statistical analysis

The statistical comparisons between groups were performed using either the t-test or the one-way analysis of variance (ANOVA). Statistical analysis of the data was performed using the SigmaStat 2.0 software (SPSS Science Inc., Chicago, IL, USA).

## Supporting information

S1 Fig Analysis of myocilin expression in human tissues by Western immunoblot using a commercial anti-myocilin antibody. (A and B) Aliquots of samples analyzed in Fig 1 (20 μg total protein/tissue extract) and 40 μg of human plasma were analyzed by SDS-PAGE (10% polyacrylamide). Conditioned culture medium from HEK-293T cells containing recombinant human myocilin (rMyoc) (25 μl) and 0.5 μg of HPLC purified recombinant myocilin (prMyoc) were used as positive controls. (A) Myocilin was detected using a purified commercial monoclonal primary antibody at a 1:50 dilution. To minimize nonspecific signals in lymphoid tissues and plasma a highly cross-absorbed anti-mouse IgG F(ab')2 fragment was used as secondary antibody (1:500). (B) As a negative control, a replica of the membrane was incubated in parallel only with the secondary antibody. MWM: molecular weight marker. Exposure time: 60 min. (C and D) Ponceau S staining of membranes showed in A and B. MWM: molecular weight marker. rMyoc: recombinant myocilin. prMyoc: purified recombinant myocilin. MWM and unlabeled lanes were not included in panels A and B.
(TIF)

S2 Fig Ponceau S staining of membranes shown in Fig 1. After Western blotting nitrocellulose membranes shown in Fig 1 were stained with Pounceau to check the amount of transferred protein. HPLC purified recombinant human myocilin (0.5 μg) was used as a positive control (prMyoc). MWM: molecular weight marker. MWM and unlabeled lanes were not included in Fig 1.
(TIF)

S3 Fig Location within the myocilin polypeptide chain of tryptic peptides identified by MALDI-TOF analysis. The peptides were obtained by in-gel trypsin digestion of Pool 2 (Fig 4C and 4D) and identified by MALDI-TOF analysis. The complete amino acid sequence of myocilin is shown. The colored boxes indicate MALDI-TOF peptides which are predicted to result from missed cleavage of none (red), one (yellow) or two (green) trypsin target peptide bonds. The identified peptides cover 64% of the myocilin amino acid sequence.
(TIF)

S4 Fig Unprocessed original scans of myocilin detection in human blood serum and plasma by Western blotting shown in Fig 3. Nitrocellulose membranes were incubated with either an anti-myocilin C21A polyclonal antibody (A) or the corresponding preimmune antibody (B). (C and D) Ponceau S staining of membranes shown in panels A and B, respectively. Exposure time: 1 min. MWM: molecular weight marker (prestained protein molecular weight marker, Thermo Scientific). The MWM lane was not included in Fig 3.
(TIF)

S5 Fig Western blot detection of myocilin present in chromatographic fractions from Fig 4A. Unprocessed original scans of myocilin. Aliquots of chromatographic fractions were analyzed by SDS-PAGE and the presence of myocilin was determined by Western blot using an anti-myocilin C21A polyclonal antibody. Exposure time: 1 min. MWM: molecular weight marker (prestained protein molecular weight marker, Thermo Scientific). The MWM lane was not included in Fig 4B.
(TIF)

S6 Fig SDS-PAGE analysis of chromatographic fractions 5 and 6 from Fig 4C. Proteins were detected by Coomassie blue staining. To maximize band separation the electrophoresis was run until the 48 kDa marker reached the bottom of the gel. MWM: molecular weight marker (prestained protein molecular weight marker, Thermo Scientific). The MWM lane was not included in Fig 4D.
(TIF)
