# Supplementary figures and images for: Identification of myocilin as a blood plasma protein and analysis of its role in leukocyte adhesion to endothelial cell monolayers
Source: PLoS One. 2018 Dec 17;13(12):e0209364. doi: 10.1371/journal.pone.0209364 (PMC6296516; doi:10.1371/journal.pone.0209364)

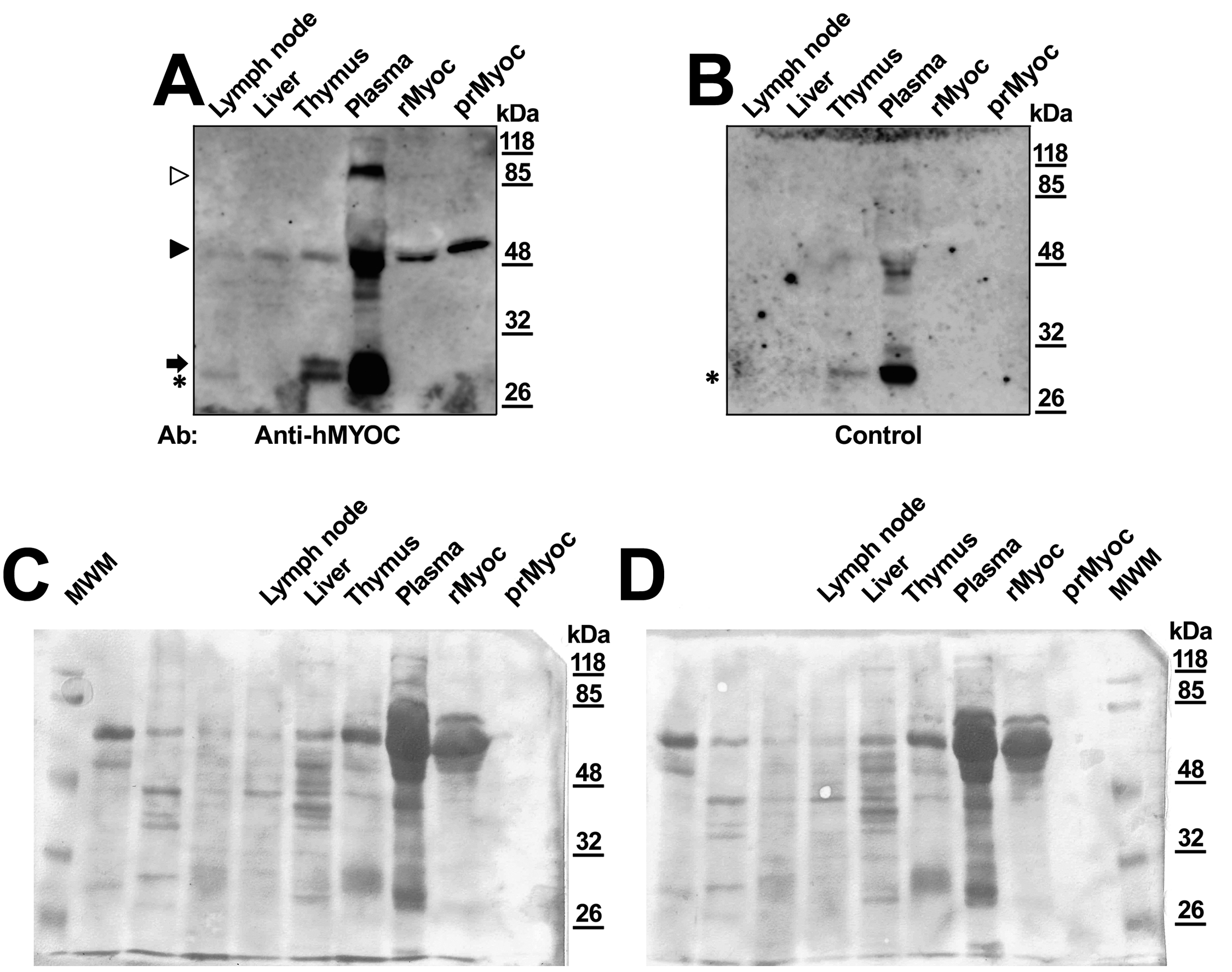

Supplement: S1 Fig — (A and B) Aliquots of samples analyzed in Fig 1 (20 μg total protein/tissue extract) and 40 μg of human plasma were analyzed by SDS-PAGE (10% polyacrylamide). Conditioned culture medium from HEK-293T cells containing recombinant human myocilin (rMyoc) (25 μl) and 0.5 μg of HPLC purified recombinant myocilin (prMyoc) were used as positive controls. (A) Myocilin was detected using a purified commercial monoclonal primary antibody at a 1:50 dilution. To minimize nonspecific signals in lymphoid tissues and plasma a highly cross-absorbed anti-mouse IgG F(ab')2 fragment was used as secondary antibody (1:500). (B) As a negative control, a replica of the membrane was incubated in parallel only with the secondary antibody. MWM: molecular weight marker. Exposure time: 60 min. (C and D) Ponceau S staining of membranes showed in A and B. MWM: molecular weight marker. rMyoc: recombinant myocilin. prMyoc: purified recombinant myocilin. MWM and unlabeled lanes were not included in panels A and B. (TIF) [file pone.0209364.s001.tif]

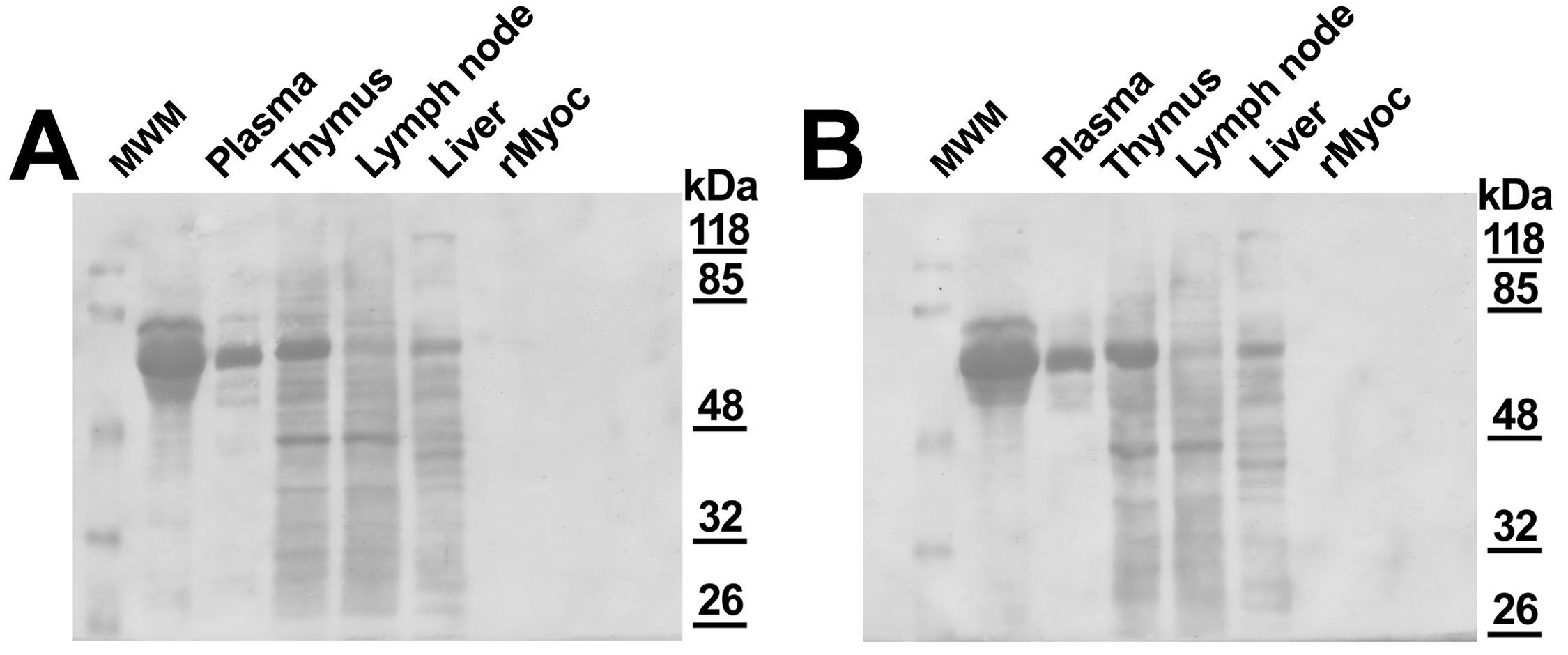

Supplement: S2 Fig — After Western blotting nitrocellulose membranes shown in Fig 1 were stained with Pounceau to check the amount of transferred protein. HPLC purified recombinant human myocilin (0.5 μg) was used as a positive control (prMyoc). MWM: molecular weight marker. MWM and unlabeled lanes were not included in Fig 1. (TIF) [file pone.0209364.s002.tif]

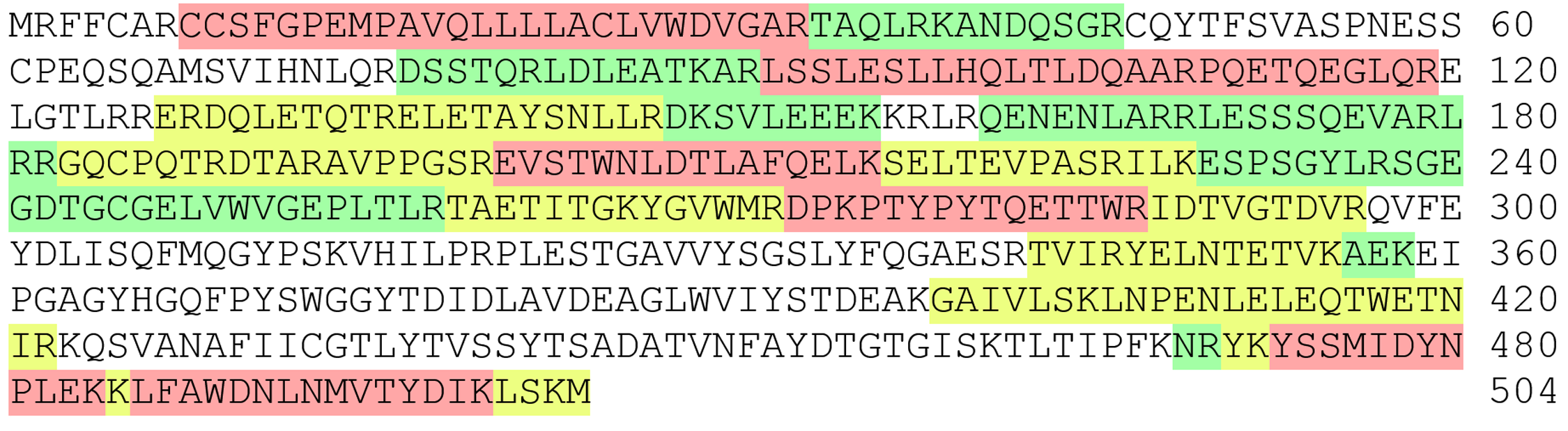

Supplement: S3 Fig — The peptides were obtained by in-gel trypsin digestion of Pool 2 (Fig 4C and 4D) and identified by MALDI-TOF analysis. The complete amino acid sequence of myocilin is shown. The colored boxes indicate MALDI-TOF peptides which are predicted to result from missed cleavage of none (red), one (yellow) or two (green) trypsin target peptide bonds. The identified peptides cover 64% of the myocilin amino acid sequence. (TIF) [file pone.0209364.s003.tif]

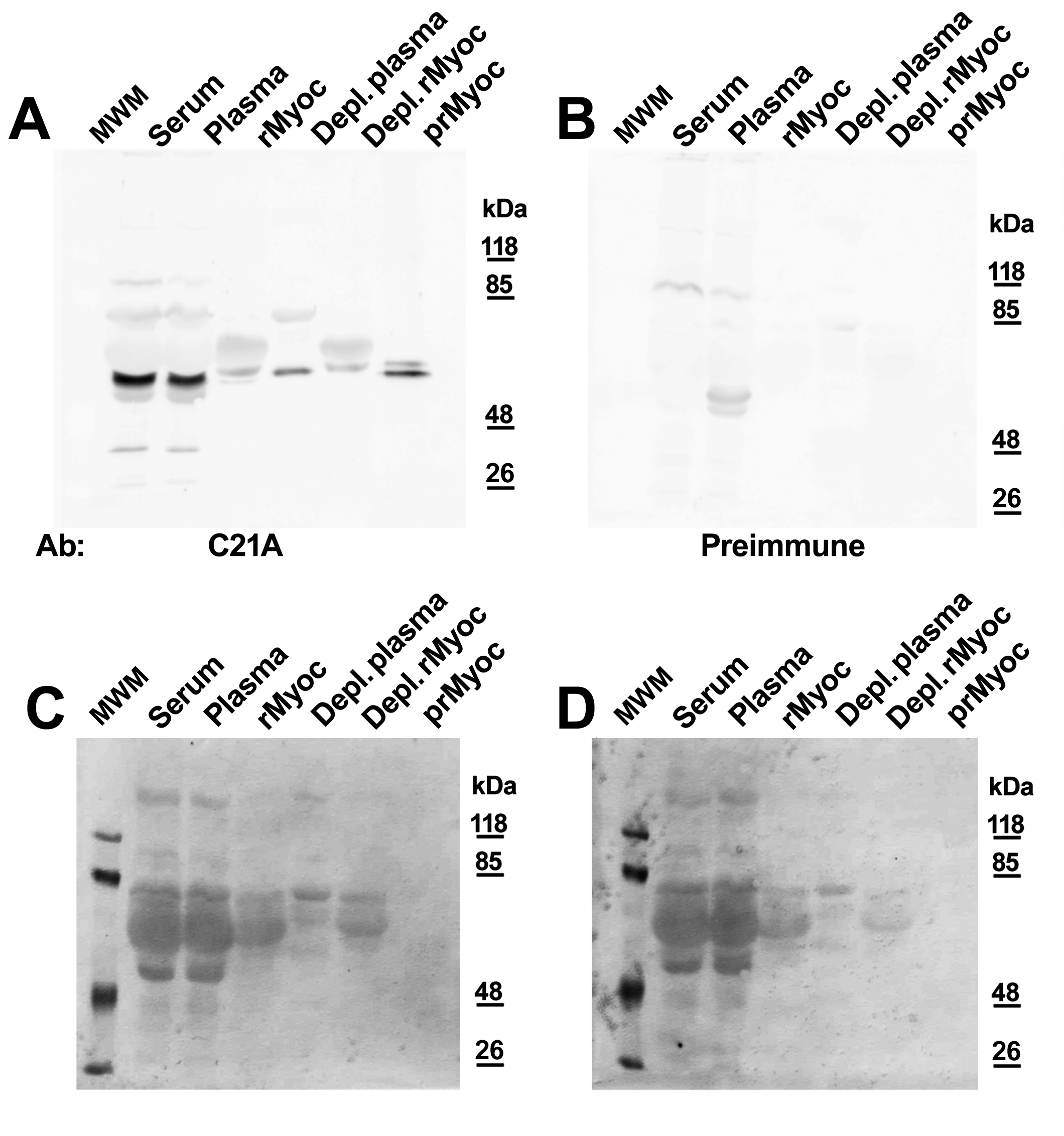

Supplement: S4 Fig — Nitrocellulose membranes were incubated with either an anti-myocilin C21A polyclonal antibody (A) or the corresponding preimmune antibody (B). (C and D) Ponceau S staining of membranes shown in panels A and B, respectively. Exposure time: 1 min. MWM: molecular weight marker (prestained protein molecular weight marker, Thermo Scientific). The MWM lane was not included in Fig 3. (TIF) [file pone.0209364.s004.tif]

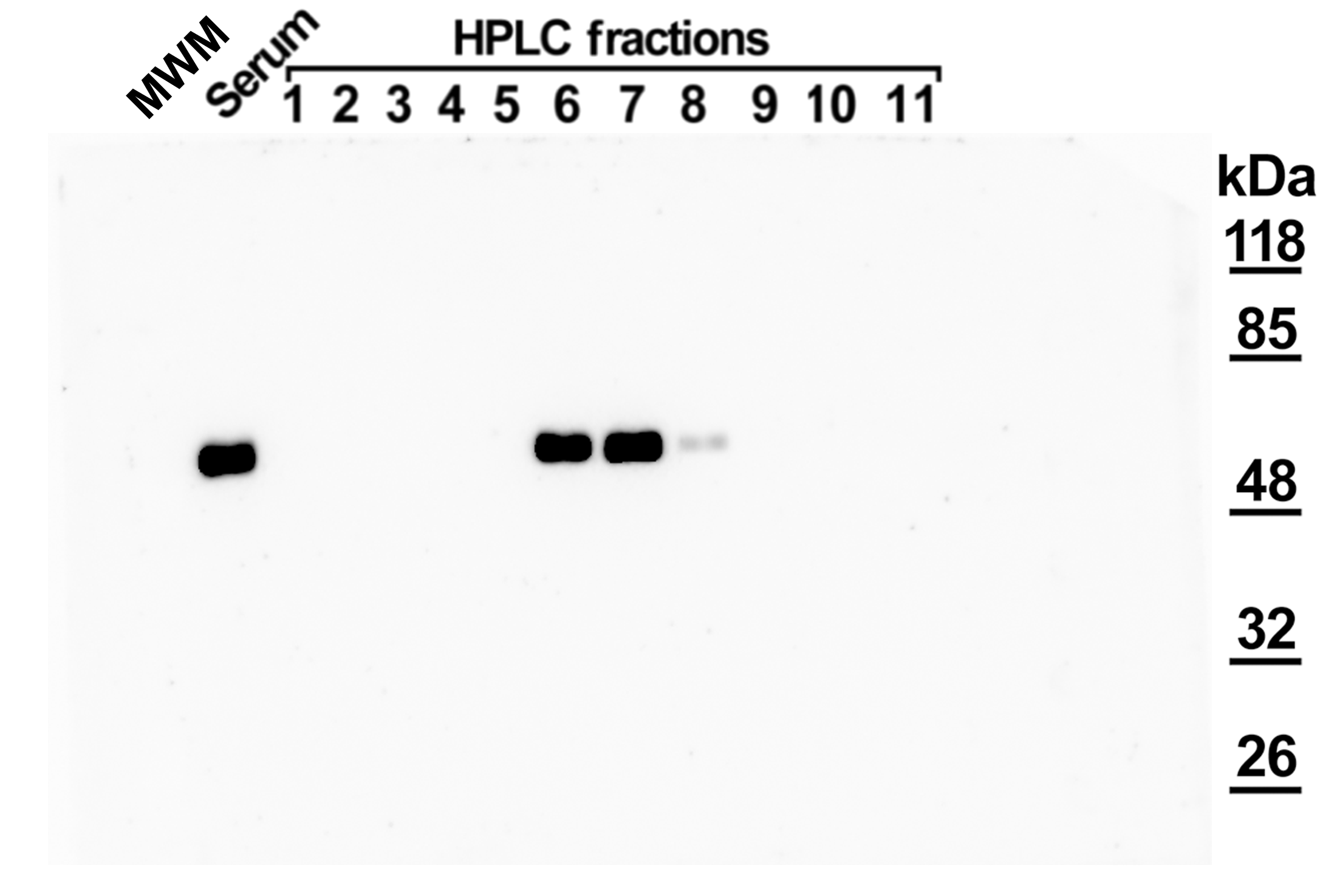

Supplement: S5 Fig — Unprocessed original scans of myocilin. Aliquots of chromatographic fractions were analyzed by SDS-PAGE and the presence of myocilin was determined by Western blot using an anti-myocilin C21A polyclonal antibody. Exposure time: 1 min. MWM: molecular weight marker (prestained protein molecular weight marker, Thermo Scientific). The MWM lane was not included in Fig 4B. (TIF) [file pone.0209364.s005.tif]

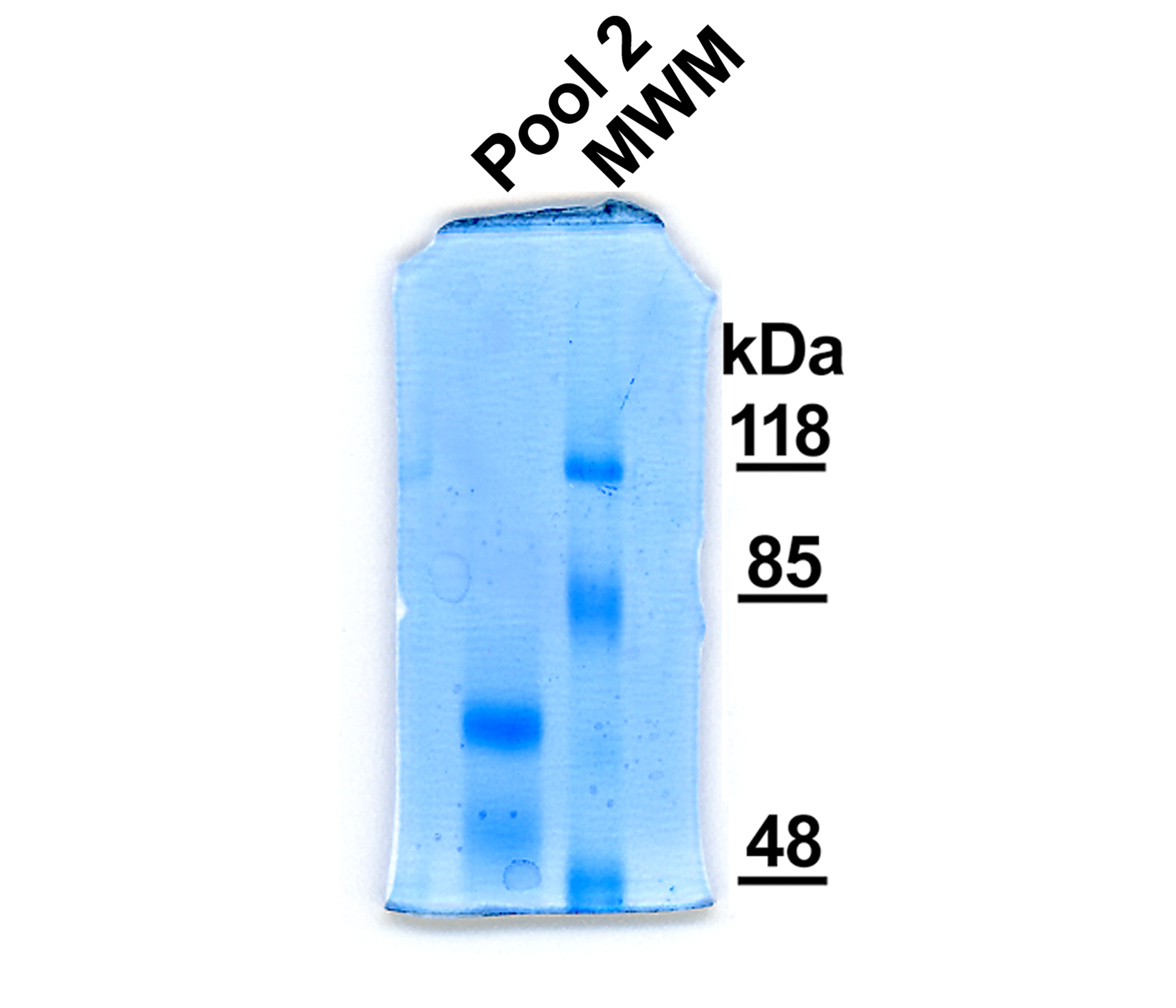

Supplement: S6 Fig — Proteins were detected by Coomassie blue staining. To maximize band separation the electrophoresis was run until the 48 kDa marker reached the bottom of the gel. MWM: molecular weight marker (prestained protein molecular weight marker, Thermo Scientific). The MWM lane was not included in Fig 4D. (TIF) [file pone.0209364.s006.tif]
